# While allied health students prefer face-to-face clinical placement, telehealth can support competency development: results from a mixed-methods study

**DOI:** 10.3389/fmed.2023.1151980

**Published:** 2023-05-15

**Authors:** Rachel Bacon, Sian Hopkins, Ekavi Georgousopoulou, Irmina Nahon, Catherine Hilly, CaraJane Millar, Allyson Flynn, Linda Smillie, Sarah Chapman, Nicholas Brown

**Affiliations:** ^1^Faculty of Health, University of Canberra, Bruce, ACT, Australia; ^2^College of Health and Biomedicine, Victoria University, Melbourne, VIC, Australia; ^3^Allied Health Clinical Education Unit, Canberra Health Services, Garran, ACT, Australia

**Keywords:** health, telehealth, clinical education, student placement, COVID-19

## Abstract

**Introduction:**

Student clinical placements are a mandatory requirement within most accredited health programs. During the COVID-19 pandemic, many health settings that had traditionally provided placements cancelled their offerings. Telehealth services however, increased and emerged as an alternative placement setting.

**Aim:**

To compare the learning experiences for allied health students provided by telehealth and face-to-face accredited health placements.

**Methods:**

Health students, from a university clinic between March to December 2020, delivering both face-to-face and telehealth consultations, were invited to complete a telephone survey with 3 demographic questions; and 10-items comparing their telehealth and face-to-face learning experiences. Pearson’s chi-squared/Fisher’s exact test was used to examine the association between each item and consultation setting. Qualitative survey data was thematically analysed using a descriptive approach.

**Results:**

49 students from 2 universities and 5 disciplines completed the survey. Students rated their face-to-face experiences significantly higher than their telehealth experiences across all items (all *p*-values <0.01). Across 9 items students reported positive learning experiences in both settings. Students had greater opportunities to work in a multidisciplinary team in a face-to-face setting. Four themes were generated: (1) placements can vary in quality regardless of setting; (2) telehealth can provide valuable learning experiences and support competency development; (3) enablers for telehealth placements and (4) barriers for telehealth placements.

**Conclusion:**

While telehealth can support student learning and competency development, in this study students preferred face-to-face experiences. To optimise telehealth placements consideration needs to be given to barriers and enablers such as technological issues and university curricula preparation.

## 1. Introduction

Most professionally accredited university health and medical courses mandate the inclusion of clinical placement hours within the curricula (1). Clinical placements enable students to translate theoretical knowledge into practice (2). They also provide the complex learning experiences necessary to develop and demonstrate competence as described by competency standards approved by professional accrediting authorities (3, 4). The demand for clinical placements has led to innovative models of clinical education such as university clinics (5, 6) which have been shown to support competency development and clinical placement capacity (7, 8).

Government restrictions in response to the COVID-19 pandemic caused many health services to transition towards a telehealth model for the safety of service users (9–12). Telehealth also allowed university clinics to continue operating, preventing the cancellation of student placements during the pandemic (13, 14). The widespread adoption of telehealth for student clinical placements has been described (15–18) but evidence of its learning benefits is only beginning to emerge.

Exploratory qualitative research on student perceptions of telehealth clinical placements in aged care nursing (19); rural medicine (20); and community allied health services (21–23); report increased clinical and communication knowledge, skills, and confidence; a greater appreciation of telehealth services; and improved employability.

Early quantitative findings suggest educational outcomes in telehealth clinical placements may be equivalent to traditional settings. Simulation research, using a randomised cross-over design (*n* = 41), found no significant difference between the diagnostic reasoning assessment scores (*t* = 0.54, *p* = 0.588) and ability to make a correct diagnosis (*t* = 0.22, *p* = 0.823) for nurse practitioners in telehealth and face-to-face standardised patient encounters (24). Similarly, Patterson et al. (25) found senior medical students perceived no significant difference (*H* = 0.0242, *n* = 26, *p* = 0.87627) in the degree of usefulness for learning from face-to-face consultations verses teleconsultations.

In allied health, however, there is less convincing evidence comparing telehealth and face-to-face placement experiences. In a recent rapid review, only 3 studies were identified (26). These had inconsistent methodologies and presented pilot data (*n* ≤ 6) or a single telehealth encounter (27–29). Positive preliminary comparisons by students (*n* = 13) on their learning experiences from an interprofessional diabetes clinic post their transition to telehealth have also recently been published (30). Their focus, however, was on interprofessional education competencies, rather than discipline specific professional accreditations requirements. This study aims to determine the difference, if any, in learning experiences for allied health students who participated in telehealth compared to face-to-face consultations within accredited health placements.

## 2. Materials and methods

### 2.1. Research setting

This research was conducted in an urban university clinic that has ten different allied health services including: a cancer wellness centre, counselling, exercise physiology, nutrition and dietetics, occupational therapy, optometry, physiotherapy, psychology, and speech pathology. The students involved in the clinics are at varying stages in their degree and placement program, ranging from first year undergraduate students to final year masters students. Telehealth was introduced in March 2020 in select clinics in response to government restrictions during the COVID-19 pandemic. This was intended to provide continuing services to the community that were safe for clinical education staff, students, and clients. As restrictions eased from July 2020, face-to-face services were gradually reintroduced, with most disciplines offering a mixture of telehealth and face-to-face services depending on best practice evidence, risk management, and client preference. Telehealth experiences within the clinic included telephone calls for screening and/or treatment of clients and video conferencing using Coviu^©^ (31) and Physitrack^®^ (32) for assessment, intervention, and discharge. Student preparation for telehealth varied across disciplines and due to stage of telehealth implementation within the health clinics.

### 2.2. Research design

This research has a pragmatist ontology, where knowledge and ideas are acquired for the purpose of solving practice-based problems (33). As such, it is the research question that has determined the method used (34). This study asks, “Is there a difference in how allied health students rate and describe their clinical learning experiences provided by telehealth and face-to-face service delivery models within accredited health placements?” A convergent mixed-methods approach was adopted to allow a more complete understanding of the phenomenon allowing the student’s voice to be heard (35). The quantitative and qualitative data are presented separately in the results and interpreted together in the discussion.

### 2.3. Procedure

Students were eligible to participate in the study if they had completed a clinical placement at the university clinic between March and December 2020. Eligible students had conducted both face-to-face and telehealth consultations whilst at the clinic. Five disciplines (exercise physiology, nutrition and dietetics, occupational therapy, physiotherapy, and speech pathology) were chosen for inclusion in the study as they provided telehealth services. All eligible students were invited to participate in the study via email with an attached participant information sheet. A member of the research team provided a follow up telephone call to give participants an opportunity to seek further clarification about the study. If consent was provided, researchers (RB, SH, IN, AF, CH, and NB) then read a script outlining the purpose and ethical considerations of the research and proceeded with the telephone survey. Researchers met prior to conducting the telephone survey to discuss the data collection process. Due to pragmatic limitations, responses could not be voice-recorded, however, researchers were instructed to document a straightforward description of the participant’s responses capturing their words as closely as possible.

A retrospective telephone survey was used to optimise the response rate (36). The survey instrument was informed by a validated student satisfaction questionnaire (37). Ten statements about student learning experiences were included to align with the research question (see Table 1). For each statement two responses were requested, one for telehealth and one for face-to-face: quantitative data was collected using a five-point Likert scale (1 = strongly agree; 5 = strongly disagree) and qualitative data by open-ended questions asking for further comment. Additionally, a final, open-ended question asking students to comment specifically on their telehealth clinical learning experiences concluded the survey. Open-ended comments were optional, and participants were not excluded from the study when open-ended responses were not provided. The questionnaire was scripted into a telephone survey to ensure consistency in the data collection. A filter question about telehealth exposure was added to ensure participants met the inclusion criteria. The final questionnaire was pilot tested by researchers with students on placement at the clinic who did not meet the inclusion criteria and modification made to improve readability.

**Table 1:** Student learning experiences of telehealth and face-to-face consultations rated using a five-point Likert scale.

Statement	Strongly agree/agree		Strongly disagree/disagree		Difference	Significance
	Telehealth	Face-to-face	Telehealth	Face-to-face		
I received adequate information about my responsibilities/expectations.	44 (89.8)	48 (98.0)	0 (0.0)	1 (2.0)	1 (2.0)	0.002
I was provided with experiences that were appropriate for a student of my background and experience.	43 (87.8)	47 (96.0)	1 (2.0)	0 (0.0)	−1 (−2.0)	0.000
I was provided with adequate opportunity to develop my competencies.	40 (83.4)	46 (95.9)	3 (6.3)	2 (4.2)	−1 (−2.1)	0.003
I was provided with a quality placement that provided a range of tasks and experiences.	40 (83.3)	45 (93.8)	1 (2.1)	0 (0.0)	−1 (−2.1)	0.000
I had appropriate learning experiences to assist me to meet my assessment requirements.	40 (87.0)	44 (95.6)	1 (2.2)	0 (0.0)	−1 (−2.2)	0.000
I felt prepared for the tasks expected of me.	37 (77.1)	44 (91.7)	5 (10.4)	1 (2.1)	−4 (−8.3)	0.000
My experiences assisted me to develop clinical problem-solving skills.	45 (93.8)	47 (97.9)	2 (4.2)	0 (0.0)	−2 (−4.2)	0.002
My experiences inspired me to develop my communication and counselling skills.	42 (85.7)	48 (97.9)	3 (6.1)	1 (2.0)	−2 (−4.1)	0.003
My understanding of the clinical practice area has improved.	44 (89.8)	49 (100)	2 (4.1)	0 (0.0)	−2 (−4.1)	0.003
I had opportunities to work in a multidisciplinary team.	13 (26.5)	36 (73.5)	24 (49.0)	6 (12.2)	−18 (−36.8)	0.010

### 2.4. Data analysis

The quantitative survey data was analysed in SPSS Version 26 using descriptive statistics. Questions that received quantitative responses to both telehealth and face-to-face components of the question were included in the data analysis. The ratings of agree/strongly agree and disagree/strongly disagree were combined to provide an overall picture of positive or negative ratings of telehealth and face-to-face experiences. Categorical data were summarised using frequencies and relative frequencies. Chi-square test (or Fisher’s exact test when necessary) was used to examine the association between responses to each statement and placement type (38). Qualitative data from the survey was collectively analysed by reflexive thematic analysis (RTA) (39) using a qualitative descriptive approach (40). SH, a novice qualitative researcher, and RB, who had experience in qualitative research, clinical education, and telehealth, both independently analysed the data through a process of data emersion, code generation and pattern recognition to construct preliminary themes. To support a process of reflexivity and increase confirmability, these researchers then met to discuss and critique each other’s interpretations, articulating their perspectives, identifying their assumptions, and learning from each other’s observations. CM also conducted an inquiry audit, increasing the dependability of the research process. SH then generated her final central organising themes.

## 3. Results

**Figure 1:**
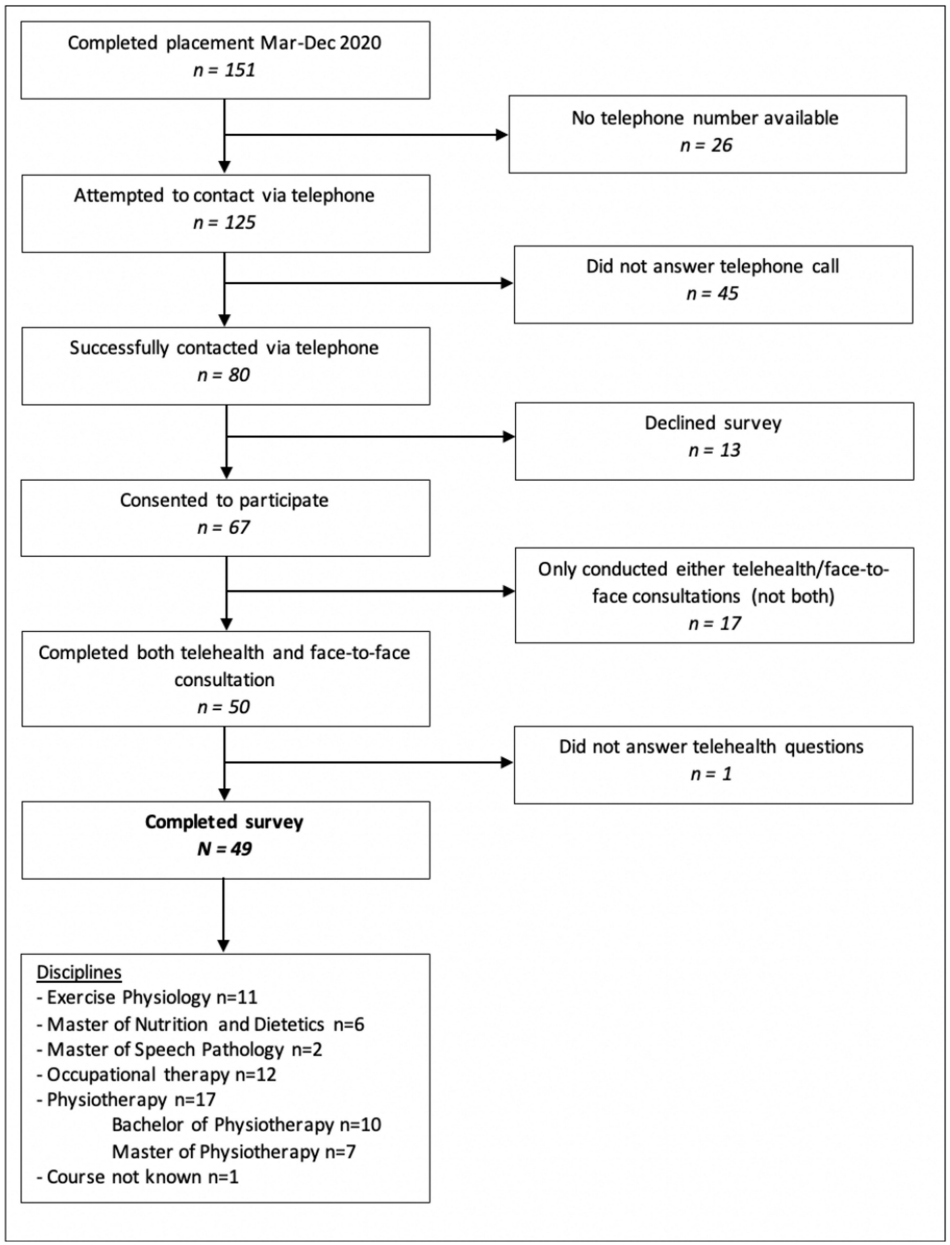
Flow diagram of student eligibility and participation in telephone surveys.

A flow diagram of the participant recruitment process and summary of the number of students from each discipline is shown in Figure 1. A total of *n* = 151 eligible students were identified, 67 consented to participate (44% response rate), and *n* = 49 students were included in the final data analysis. The number of students from each discipline is as follows: Exercise Physiology (*n* = 11), Master of Nutrition and Dietetics (*n* = 6), Master of Speech Pathology (*n* = 2), Occupational Therapy (*n* = 12), and Physiotherapy (*n* = 17). Students from two Universities participated in placements at the university clinic and were included in the study: University one (*n* = 42) and University two (*n* = 5). Students completed either an even mix of face-to-face and telehealth consultations (*n* = 14), predominantly face-to-face with some telehealth (*n* = 26), or predominantly telehealth with some face-to-face (*n* = 9).

### 3.1. Quantitative results

Most students agreed or strongly agreed with nine statements for both telehealth (>77.1% agree/strongly agree) and face-to-face (>91.7% agree/strongly agree), however, face-to-face was rated significantly higher on all ten statements (all *p*-values <0.01). On the final statement “I had opportunities to work in a multidisciplinary team,” students stated that they had significantly more opportunities when conducting face-to-face consultations (73.5% agree/strongly agree, 12.2% disagree/strongly disagree) compared to telehealth (26.5% agree/strongly agree, 49.0% disagree/strongly disagree; *p* = 0.003). A summary of the quantitative results for all ten statements is shown in Table 1.

### 3.2. Qualitative results

Approximately half the participants provided responses to the open-ended questions [∑ = 293, *x* = 27 (54%)], with the majority (73%) responding to the final open-ended question. Four themes were generated by the researcher SH from the participants responses to the open-ended questions. These are: (1) placements can vary in quality regardless of setting; (2) telehealth can provide valuable learning experiences and support competency development; (3) enablers for telehealth placements; and (4) barriers for telehealth placements.

#### 3.2.1. Placements can vary in quality regardless of setting

Participants reported that their experience of a telehealth placement can be influenced by a variety of factors, many of which are not unique to telehealth such as client numbers and case-mix,

“*I feel like on the placement I had, we saw a huge range of different clients, who all had varying backgrounds, conditions etc. This allowed myself, and other students a huge amount of growth and exposure to different clients which was great.*” *(Q4, Exercise physiology student).*

“*The range of clients was not much. We got people coming in for the same thing which got quite boring.” (Q4, Occupational therapy student).*

learning opportunities available during the placement,

“*Even in my first week, my supervisor was open to us taking learning opportunities. We had the opportunity to observe but we could also have a go ourselves. We were always offered the challenge to extend our learning. Yes—we always had a say.*” *(Q3, Nutrition and dietetics student).*

“*I had opportunity to work closely with speech pathology and once or twice during my placement time was had a case study where our supervisors gave us the information about a client and as a multidisciplinary team came together to discuss this client. We had different perspectives and I had opportunities to learn about how the team takes the client from their perspectives. It was helpful and was an interesting experience.*” *(Q3, Occupational therapy student).*

and the student’s stage of placement.

“*I found telehealth difficult, interacting with the client being a first placement. I could see that students who had more experience were more confident and were more comfortable in that setting. If it was the last placement, it would be a better experience but as it was my first placement it was harder.*” *(Q11, Occupational therapy student)*.

“*The only difference was the complexity of the clients. For me, my face-to-face consultations were more complex. Telehealth was a nice opportunity to gather information in a different way—to look in the pantry and make the patient feel more comfortable using their home environment.*” *(Q7, Nutrition and dietetics student)*.

Participants highlighted how the educational approach of their clinical educators impacted the quality of their placement,

“*I had two supervisors. One of them I don’t think helped me very much at all, was very critical of everyone. We were scared to ask her questions. My other supervisor was really good, we could ask her anything and get her to check our work.*” *(Q5, Exercise physiology student)*.

Participants valued a supportive environment, and regular and constructive feedback following telehealth consultations.

“*Our educator was always there for us. After each session we were provided with feedback and comments to improve our next consultation. It was really great.*” *(Q2 Nutrition and dietetics student)*.

#### 3.2.2. Telehealth can provide valuable learning experiences and support competency development

Participants talked about how when using telehealth, they developed their clinical reasoning and problem-solving skills, improved their resourcefulness and flexibility, and learned to communicate effectively in ways that are different to face-to-face experiences.

“*Telehealth placed a larger emphasis on clinical problem solving and working in different situation and consolidated some of that learning.*” *(Q7, Physiotherapy student)*.

“*Both options gave different opportunities to meet my assessments. Telehealth required flexibility, adaptability, and skills with using technology.*” *(Q5, Exercise physiology student)*.

Participants reported increased work-readiness as they gained an additional skill set and appreciation for the benefits of telehealth to their practice. Development of communication skills stood out as a particular strength of telehealth under this theme.

“*The telehealth session, it was working well for the client’s family. The client didn't have to come to the session, and they could do it at home so there was no travelling time. The parents were more involved in the session with the child, so the parents have more opportunity to practice at home whereas in the (face-to-face) session the parents have to prepare in advance. …I saw lots of benefits of telehealth sessions after that.*” *(Q11, Occupational therapy student)*.

“*I think having the opportunity to use telehealth in the context of placement did provide me with an understanding of the merit of using telehealth as a practitioner in the future and I think that by using telehealth I was able to focus on my communication skills in a way that I probably would not have provided so much attention to if I had all my consultation face-to-face. I was more aware of my tone of voice, the way I phrased things, such as my diet disease relationships. I paid more attention to listening to verbal cures from clients. I feel fortunate that I got to experience this. My future career may involve using telehealth platforms and I felt fortunate that I got to experience that on my placement.*” *(Q11, Nutrition and dietetics student)*.

Participants reported that they found the hands-off nature of telehealth encouraged them to develop their verbal communications skills. This was particularly noted for participants from physiotherapy and exercise physiology, whose disciplines are usually considered “hands-on”.

“*Telehealth really helped my clinical reasoning skills as I could not do any hands-on skills, so I needed to develop more questioning skills to describe what I needed to know and what I needed to tell them.*” *(Q11, Physiotherapy student)*.

“*Telehealth required you to be clear and precise with your descriptors/measurements and analysis tools and you needed to work harder to make the client feel comfortable more quickly.*” *(Q8, Nutrition and dietetics student)*.

Some participants however, felt that a telehealth placement did not support their development in all areas of practice.

*The face-to-face was more applicable to our university workload. We had practical assessments—we had more opportunity to develop the ‘hands-on’ tasks (Q5, Exercise physiology student)*.

Telehealth provided an opportunity for safe delivery of placements during the COVID-19 pandemic and for innovation and engagement in the clinic during placements. For example, participants studying physiotherapy had opportunities to complete projects related to telehealth that increased their learning opportunities.

“*Impressed during the telehealth placement how the supervisors got us involved in the projects. Even when we had time off there were tutorials that gave us opportunities to learn.*” *(Q4, Physiotherapy student)*.

#### 3.2.3. Enablers for telehealth placements

Participants described how positive telehealth experiences are enabled by factors such as the provision of learning scaffolds, and induction and site training that familiarises students with telehealth delivery.

“*I didn’t know anything about telehealth before placement. Everything was detailed for me about what I needed to get from the client and how to use telehealth services.*” *(Q1, Exercise physiology student)*.

“*With the telehealth one, as I’m a first-year student, we were provided with a script which really helped me.*” *(Q2, Occupational therapy student)*.

Adequate opportunity to prepare for consultations, client screening, and regular feedback from supervisors using competency-based assessment tools also enabled participants to have positive telehealth experiences.

“*Our supervisors had us create a pre-telehealth questionnaire to give to the clients. After making that I felt more prepared.*” *(Q6, Nutrition and dietetics student)*.

“*Our supervisors gave us feedback which helped us improve. There was adequate feedback given after telehealth as well.*” *(Q3, Occupational therapy student)*.

#### 3.2.4. Barriers for telehealth placements

Participants shared how their experiences of telehealth can be negatively impacted. This can be due to issues with the telehealth itself, such as sophistication of the platform or technical issues,

“*What came out of telehealth was about the quality of the platform we were using, especially for the needs of the clinician and the client. For speech pathology we play games and have children relax, with the platform we used we couldn't give control to the client to support their engagement in sessions.*” *(Q11, Speech pathology student)*.

“*Telehealth delivery was quite new. At times, we lost connection and lost momentum with the client.*” *(Q2, Exercise physiology student)*.

or due to uncontrollable factors such the challenges associated with rapid implementation of telehealth within the clinic in the context of the COVID-19 pandemic.

“*I was happy that we were able to transition to telehealth rather than cancelling the placement. We were able to work in areas that we wouldn’t in a face-to-face placement. Instructors did a good job of helping us transition and to help us through technical and other difficulties that we had during the process.*” *(Q11, Physiology student)*.

Learning related barriers were identified by the participants. This included limited inclusion of telehealth in the curricula prior to placement and the rapid adaption of face-to-face administered assessments to telehealth practice.

“*Given that we weren’t given any training in telehealth during our university studies, it was a stretch to start doing telehealth consultations only, but we were given enough training to make that transition successfully.*” *(Q2 Physiotherapy student)*.

“*I just think with telehealth my assessments weren’t really written for telehealth, so it wasn’t fully meeting my expectations.*” *(Q5, Occupational therapy student)*.

“*Sometimes the system can be easy to follow. We needed more set-up assistance for telehealth. We needed more practice. My hours were lower than some others, but I need to know more about how to be professional and to do things better over telehealth.*” *(Q3, Exercise physiology student)*.

## 4. Discussion

This mixed methods study contributes to the body of evidence on the educational contribution of telehealth to allied health student clinical placements. Unlike Posey et al. (41) and Patterson et al. (25) our quantitative results showed a statistically significant difference between telehealth and face-to-face placement learning experiences across all items. Yet, like Patterson et al. (25) while our students preferred face-to-face consultations, they found both settings provided useful learning experiences. Most participants in our study agreed or strongly agreed that telehealth experiences provided high quality learning experiences (Statement 4), were aligned with the assessment requirements (Statement 5), assisted them to develop their competencies (Statement 3); enabled the development of clinical problem-solving skills (Statement 7), and improve their clinical practice (Statement 9).

Our qualitative results help us to understand these results. Qualitative comments from participants in our study suggested that the stage of placement influenced their perception of telehealth learning experiences. Posey et al. (41), found a statistically significant difference related to the sequence of encounter type, with students more likely to make a correct diagnosis when the first encountered patients were face-to-face rather than telehealth (*x*^2^ = 9.7, *p* < 0.01). They suggest that it may in part be due to the lack of familiarity with the technology. In line with previous research (19–21, 42), our study identified technical issues related to telehealth as a barrier to positive learning experiences during telehealth consultations. For example, Shortridge et al. (43) found that complaints about technical issues in student reflections coincided with lower scores on a Telehealth Acceptance Survey following telehealth clinical activities. Offering skills training to clinical educators and students on the telehealth technology prior to conducting telehealth consultations may mitigate this concern. A 2021 review on telemedicine in the medical curricula identified that students would benefit from additional time to become familiar with and practice using telehealth technologies (44). Given the rapid rollout of telehealth during COVID-19, like other students, students and the clinical educators implemented telehealth on the run and learned how to prepare students on subsequent placements (17, 19, 22, 45).

The quality of a clinical placement is dependent on many factors (46–48), independent of whether the placement is completed via telehealth or face-to-face. For example, this can be due to the students’ interactions with their clinical educators including the educators’ telehealth skills, teaching styles, progress of telehealth implementation in the clinic, and student preparation for placement (49). Other factors that influence student experiences may also be individual, such as the student’s prior skills and abilities, physical and emotional state, attitude, learning styles or personality traits (50). In this study, the same students in the same placement settings rated both their face-to-face and telehealth experiences, minimising these variables. The rapid rollout of telehealth in the context of a pandemic, however, must be considered in interpreting these results. This influenced client numbers and case-mix, the learning environments, and the student’s interactions with their supervisors.

As found by other researchers (16, 22–24), some of our participants expressed concern that telehealth did not support their development in all areas of practice, particularly for ‘hands-on’ tasks. Yet, telehealth also offered some unique advantages. Participants reported increased resourcefulness and flexibility, greater appreciation and capability with telehealth, and improved communication and professional skills. Pelly et al. (51) found similar results, arguing that such capabilities are well aligned with future workforce needs. The Australian Government’s recent decision to continue subsidising telehealth (52) reinforces that telehealth is now a mainstream health service and its delivery is an essential capability for health graduates. Some disciplines have introduced new competency standards in telehealth (53) while others advocate for more generic competency standards aligned with its delivery (54). Certainly, consistent with our findings, there is a call to embed telehealth into the health curricula (10, 19–22, 26, 44, 55–57).

In our study participants reported fewer opportunities to work in a multidisciplinary team (Statement 10), although it is not clear if this is a limitation of the university clinic rather than telehealth itself. The rapid shift from face-to-face to telehealth in response to the COVID-19 pandemic may have resulted in a move away from the structures that were in place at the clinic for multidisciplinary learning experiences. Previous research on interprofessional education and telehealth has focused on programs structured specifically for interprofessional telehealth activities (43, 58, 59). It may be that a specific focus on providing multidisciplinary or interprofessional learning experiences is needed to enable this to take place within university clinics. Telehealth offers unique capabilities ideally suited to interprofessional care delivery (60), with recent preliminary (*n* = 13) evidence from Pittman et al. (30) which showed that Total Team Skills Scale scores (a self-assessment measure of interprofessional team skills) improved significantly after participating in the telehealth service (*Z* = 2.9, *df* = 12, *p* = 0.004).

### 4.1. Limitations

Despite its novelty and currency, this study has some limitations. The number of placements that each student had undertaken prior to their placement at the university clinic was not recorded, nor was their level of experience in using telehealth. It should be noted, however, that 87% of students reported to have been provided with experiences that were appropriate given their prior learning (Statement 2). Some of the disciplines had only recently established their clinics within the university clinic and had a small number of students completing placements during the study period. This meant that it was not possible to make a statistical comparison of quantitative responses to determine whether results varied by student experience or between disciplines. We did not measure the time taken for students to complete tasks or compare this time from face-to-face to telehealth. However, these limitations were strengthened by qualitative comments that provided some context to students’ responses, and an understanding of how and why student experiences differ. This study is based on retrospective participant reported data. It is recommended that further research be undertaken that measure changes in students’ learning development and outcomes after attending telehealth clinical placement experiences.

### 4.2. Conclusions and future directions

The findings of this study have important implications for allied health education and professional accreditation. Our study provides evidence for allied health courses across a range of disciplines (exercise physiology, nutrition and dietetics, occupational therapy, physiotherapy, and speech pathology) for the use of telehealth to develop and assess students’ professional competence as part of an overall placement program. As concluded by other researchers (22, 24), telehealth is an ‘*important tool in the toolkit’* [Ross et al. (22), p. 14] in student clinical placement education. This research calls for the integration of telehealth into the health curricula including the development of communication skills and interprofessional experiences specifically for telehealth, and onsite support and training with telehealth technologies. There is a need for more robust research on allied health telehealth clinical placement experiences and competency development, particularly without the added complexities of the COVID-19 pandemic.

## Data availability statement

The original contributions presented in the study are included in the article/supplementary material, further inquiries can be directed to the corresponding author.

## Ethics statement

The studies involving human participants were reviewed and approved by the University of Canberra Committee for Ethics in Human Research (CEHR 4431). The participants provided their written informed consent to participate in this study.

## Author contributions

RB, EG, SH, AF, IN, CM, CH, NB, LS, and SC contributed to the research design, funding application, and data collection. RB completed the ethics application. SH, EG, and RB completed the initial data analysis. SH and RB drafted the manuscript. All authors contributed to the article and approved the submitted version.

## Funding

This research was funded by an Australian Collaborative Education Network (ACEN) Research Grant 2020.

## Conflict of interest

The authors declare that this research was conducted in the absence of any commercial relationships that could be constructed as a potential conflict of interest.

## Publisher’s note

All claims expressed in this article are solely those of the authors and do not necessarily represent those of their affiliated organizations, or those of the publisher, the editors and the reviewers. Any product that may be evaluated in this article, or claim that may be made by its manufacturer, is not guaranteed or endorsed by the publisher.
